# Immature stages and ecology of two species of the South African genus *Stripsipher* Gory & Percheron, 1833 (Coleoptera, Scarabaeidae, Cetoniinae, Trichiini)

**DOI:** 10.3897/zookeys.180.2315

**Published:** 2012-04-05

**Authors:** Petr Šípek, Enrico Ricchiardi, Renzo Perissinotto

**Affiliations:** 1 Department of Zoology, Faculty of Science, Charles University in Prague, Viničná 7, 128 44 Praha 2, Czech Republic; 2 Corso A. Tassoni 79/4, 10143 Torino, Italy; 3 School of Life Sciences, University of KwaZulu-Natal, Westville Campus, P. Bag X54001, Durban 4000, South Africa

**Keywords:** Trichiini, *Stripsipher*, New Synonym, South Africa, Immature stages, Larva, Pupa

## Abstract

Based on the study of newly accessible type material, *Stripsipher drakensbergi* Ricchiardi, 1998, is demoted to a junior synonym of *Stripsipher jansoni* Péringuey, 1908. The genus *Stripsipher* Gory & Percheron, 1833, thus, currently includes 12 species, but for none of these are larval stages and/or pupae currently known. The immature stages of *Stripsipher orientalis* Ricchiardi, 2008 and *Stripsipher jansoni* are described here for the first time and updated observations on distribution and ecology of both species are provided. Morphological affinities of *Stripsipher* with other Trichiini larvae are presented and the main diagnostic differences discussed. The larvae of both species are very similar to those of other representatives of the tribe Trichiini, with key differences found on the epipharynx. Based on the morphology of larvae and adults, it is suggested that *Stripsipher* is a member of the clade composed of Valgini, Trichiini and Cryptodontini.

## Introduction

Following the latest revision, the genus *Stripsipher* currently includes 13 species (Ricchiardi et al. 2008) (see list below). For one of these, *Stripsipher jansoni* Péringuey, 1908, it had not been possible in the past to examine any type specimen and, therefore, the position of *incertae sedis* had been preferred for the species. Recently, the lectotype (here designated) has become accessible for analysis, courtesy of J Krikken of the Museum Naturalis, Leiden. Péringuey (1908) described this species from a pair, however the actual location of the female is unknown. This has now revealed that *Stripsipher jansoni* is actually a senior synonym of *Stripsipher drakensbergi* Ricchiardi, 1998. Therefore the current number of recognized species for the genus is reduced to 12.

An important contribution to this analysis could come from studies of larval characteristics of the species included in the genus. Unfortunately, there are still too few descriptions of Trichiini larvae and none currently known for the genus *Stripsipher*. Here, we provide the first description of the larvae of two species of this genus, which were conclusively identified through the rearing of cohorts collected in their natural environments.

## Material and methods

The classification of the Cetoniidae used here follows Krikken (1984). A number of divergent classification systems have been proposed recently (e.g. Scholtz and Grebennikov 2005; Smith in Bouchard et al. 2011), but they remain equally controversial and not universally accepted.

The terminology for larval morphology follows Hayes (1929), Böving (1936), Ritcher (1966) and Sawada (1991). Hair-like setae of cranium and other structures were classified according to their relative size into two groups, medium to long (100–500 μm) and minute (20–50 μm, or less) setae, in order to give the most accurate information on chaetotaxy. Refer to Šípek et al. (2008; Fig. 12) for a detailed schematic figure. Morphological analysis and measurements were carried out using Olympus SZX9 and Olympus BX 40 light microscopes, both equipped with digital camera Olympus Camedia 5060. Mouthparts were dissected and if necessary mounted on slides in Liquide de Swan (e.g. Švácha and Danilevsky 1986). Photographs of beetles and larvae were taken using a Canon 550D digital camera equipped with a Canon MP-E 65/2,8 MACRO lens with 5:1 optical magnification. Final images were composed from multiple layer-focus pictures using Helicon Focus software. Structures examined at the scanning electron microscope JEOL 6380 were cleaned in 10% lactic acid for 24 hours, dried with critical point drying and mounted on aluminium plates. Drawings were made on the basis of photographs and all photographs were digitally enhanced (levels adjustment, background elimination, sharpening) using Adobe Photoshop.

### Larval rearing

Eleven and 22 larvae of *Stripsipher jansoni* and *Stripsipher orientalis* were collected at Cobham (KZN southern Drakensberg) or Karkloof and Entumeni (KZN Midlands), respectively. Three to five specimens of each population were immediately fixed in 10% formalin, while the others were reared to adulthood in the laboratory, using their natural food sources (soil detritus and decomposing wood). They were kept in an environmental control room at the University of KwaZulu-Natal at a constant temperature of 22°C during the summer, which was then gradually decreased during the colder seasons to a minimum of 15°C in winter (July–September). Larvae were kept in 10-litre buckets filled to approximately half capacity with their natural soil/wood. The surface layer of each bucket was sprayed with tap water at regular weekly intervals until pupation of the bulk of the larvae, but left to dry out thereafter. The success rate was about 80% for both species, with adults emerging in good conditions over a period of about two months, from late October to December.

### Phylogenetic analysis

To test the phylogenetic position of *Stripsipher*, a previously published larval/adult morphological dataset (Micó et al. 2008, Šípek et al. 2009) was used, which was supplemented with new data. Methods of phylogenetic reconstructions follow Šípek et al. 2009. All bioinformatic analyses were carried out on the freely available Bioportal (www.bioportal.uio.no).

### Abbreviations used

Province & country codes

**LES** Kingdom of Lesotho

**RSA** Republic of South Africa

**SWA** Kingdom of Swaziland

RSA provinces

**ECA** Eastern Cape

**FST** Free State

**GAU** Gauteng

**KZN** KwaZulu-Natal

**MPU** Mpumalanga

**NCA** Northern Cape

**NWE** North-West

**LIM** Limpopo

**WCA** Western Cape

### Collection and specimen codes

**ISAM** Iziko South African Museum, Cape Town (RSA)

**MNHN** Museum National d’Histoire Naturelle, Paris (France)

**ERC** Private Collection Enrico Ricchiardi, Turin (Italy).

**RPC** Private Collection Perissinotto & Clennell, Durban (RSA)

**RNHL** Netherlands Centre for Biodiversity, Naturalis (formerly known as National Museum of Natural History) , Leiden (Netherlands)

**SANC** South African National Collection of Insects, Pretoria (RSA)

**TMSA** National Museum of Natural History (formerly Transvaal Museum), Pretoria (RSA)

**HT** Holotype

**PT** Paratype

**LT** Lectotype

## *Stripsipher* species list

*Stripsipher braunsi* Ricchiardi, 1998 WCA, ECA

*Stripsipher centralis* Ricchiardi, 1998 KZN, FST, MPU

*Stripsipher jansoni* Péringuey, 1908 ECA, KZN, LES, FST, MPU = *Stripsipher drakensbergi* Ricchiardi, 1998 Syn. nov.

*Stripsipher lamellatus* Ricchiardi, 2008 KZN

*Stripsipher longipes* (Swederus, 1787) WCA, ECA, KZN, MPU, GAU, NWE

*Stripsipher orientalis* Ricchiardi, 2008 ECA, KZN, MPU, GAU, NWE, NOP, SWA

*Stripsipher signatulus* Ricchiardi, 2008 KZN

*Stripsipher spectralis* Arrow, 1926 ECA, KZN

*Stripsipher superbus* Ricchiardi, 2008 KZN

*Stripsipher turneri* Arrow, 1926 ECA, KZN

*Stripsipher werneri* Ricchiardi, 1998 KZN

*Stripsipher zebra* Gory & Percheron WCA

## Distribution and ecology

### Stripsipher orientalis

**Figure 1.**
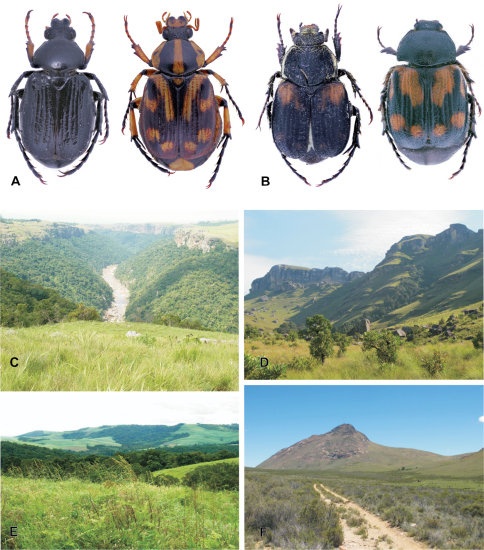
**A** *Stripsipher orientalis*: male (left, melanic form) and female (right, typical form) **B** *Stripsipher jansoni* male (left) and female (right) **C, E** dense riverine and afromontane forest pockets  are the preferred habitats of *Stripsipher orientalis* (**C** – Umthamvuna KZN **E** – Entumeni KZN) **D, F** mountain grassland habitats of *Stripsipher jansoni* (**D** – Cobham/Drakensberg KZN **F** – Compassberg ECA,). (Fig. 1C–F Lynette Clennell).

**Figure 2.**
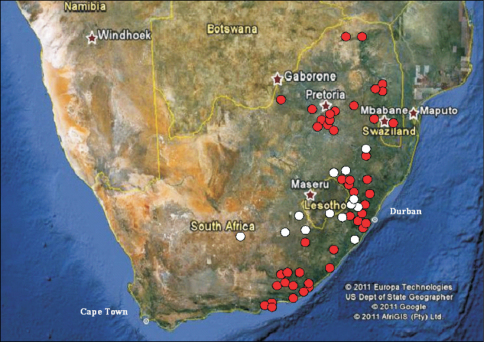
Distribution of *Stripsipher orientalis* (red circles) and *Stripsipher jansoni* (white circles).

This is a forest dwelling species (Figs 1A, C, E), which completes its larval development inside old tree trunks and branches, feeding on decaying, soft wood. It is distributed from the Eastern Cape north-eastwards, including KwaZulu-Natal, Swaziland, Mpumalanga, Gauteng, North–West and Limpopo (Fig. 2).

Larvae have been found in a variety of tree species (e.g. *Podocarpus* spp., S. Endrödy-Younga and P. Istvan, specimen data labels), mostly in relict afromontane and coastal rainforests (e.g. Fort Fordyce, Umthamvuna, Karkloof, Entumeni, Monk’s Cowl). Invariably, they attack old wood that has already been partly consumed by other saproxylic species, but is still suspended in the air or at least not directly in touch with the ground, presumably in order to avoid soil predators (e.g. ants). They appear to avoid entirely dying or recently dead trunks and branches. Larvae have been reared successfully in captivity, in environmental control rooms in Durban, by one of the authors (R.P.).

Adults of the species are active from September to February, exhibiting a peak of records during the months of December (33% of total) and November (26%). They have been observed flying in dense forests around decomposing wood during the hottest part of the day, or alternatively crawling on dead trunks/ branches and even in tunnels inside the wood. They feed on nectar, having been recorded on a variety of flower types, including *Buddlia saligna*, *Sizigium cordatum*, *Dalbergia obovata*, *Dalbergia armata* and *Protea caffra*. They have also been captured with fruit-baited traps on at least two occasions (S. Endrödy & M. Klimaszew and O. Bourquin, specimen data labels) and in Malaise traps (R. Miller, specimen data label), but not at sap flows.

**Material examined.** SOUTH AFRICA: HT♂, GAU, Johannesburg, W.G. Kobrow (ISAM); PT 1♀, GAU, Florida, 15 Oct 1976 (TMSA); PT 1♀, MPU, Graskop, Dec 1974, P.E. Reavell (TMSA); PT 1♀, KZN, Drakensberg, Cathedral Peak, 28 56 S, 29 13 E, under bark, 25 Nov 2003, M. Burger & R Müller (TMSA); PT 1♀, ECA, Transkei, Umtata, 25 Nov 1989, N. Duke (TMSA); PT 1♀, GAU, Witbank M.J.P, Dec 1961, A.R.I. Pretoria (TMSA); South Africa, KZN, 1 PT (#), New Hanover, Dec 1955, Natal A.R.I., M.B. Bayer legit (TMSA); PT 1♀, MPU, Belfast, 6 Dec 1988 (TMSA);PT 1♀, NWE, TUC, SE 2526 Ab, Zeerust, 22 Dec 1987, W.Z. Schultz (TMSA); PTs 6♀, MPU, Belfast, 25 38 S, 30 08 E, 15 Dec 1988, R.I. Mansfield (TMSA); PT 1♀, MPU, Berlin F.S. gorge, 25.32 S, 30.44 E, 23 Oct 1986, E-Y: 2304, intersept trap 42d, Endrody-Younga (TMSA); PT 1♀, KZN, Himeville, Farm Meander (Brookland), 29 35 S, 29 42 E, Dec 1988, S. Mclean (TMSA); PT 1♀, ECA, East London, 2 Dec 1921, H.K. Munro (TMSA); PT 2♀, MPU, Belfast, 6 Dec 1988, H.P. Terblanche (TMSA); PT 2♀, ECA, Kubusie Forest, Stutterhaim, 3 Jan 1980, N.J. Duke (TMSA); PT 1♀, ECA, next Kologha Forest, 1092 m, 6 Km NE Stutterheim, 32 33 S, 27 22 E, day beating, 25 Nov 2000, Krüger & Dombrowsky (TMSA); PT 1♀, GAU, Jan Smuts, SE 2826 Aa, 27 Nov 1980, C.L. v/d Hoven (TMSA); PT 1♀, KZN, Weza Ingeli Forest, 30.32 S, 29.41 E, E-Y: 2692, hanging fruit traps, 18 Nov 1989, Endrody & Klimaszew (TMSA); PT 1♂, KZN, Ferncliffe Forest Res, 29 33 00 S, 30 20 30 E, 975 m, Mistbelt Mixed Forest, 23 Nov 1987, J.G.H. Londt (TMSA); PT 1♂, MPU, Barberton, Miss De Beer (TMSA); PT 1♂, MPU, Belfast, 25 38 S, 30 08 E, 15 Dec 1988, R.I. Mansfield (TMSA); PTs 2♂, ECA, Kubusie Forest, Stutterhaim, 6 Jan 1982, N.J. Duke (TMSA); PT 1♂, ECA, Hogsback, 14 Feb 1978, N.J. Duke (TMSA); PT 1♂, ECA, The Haven, Transkei, 2 Jan 1981, N.J. Duke (TMSA); PT 1♂, ECA, East London: Buffalo Pass, 29 Sep 1984, N.J. Duke (TMSA); PT 1♂, KZN, Karkloof, 12 Feb 1929, Bill Marley (TMSA); PTs 5♂, GAU, Johannesburg, Emerentia, Sep 1974, H.R. Heburn (TMSA); PT 1♂, GAU, Magaliesburg, 26 00 S, 27 32 E, 16 Nov 1993, (TMSA); PT 1♂, GAU, Johannesburg, Transvaal, Nov 1931, G. Kobrow (TMSA); PT 1♂, GAU, Johannesburg, Transvaal, Nov 1934, G. Kobrow (TMSA); PT 1♂, KZN, “Northington”, Dargle, 28 27 S, 30 04 E, 25 Jan 1988, baited forest trap near edge, O. Bourquin (TMSA); PT 1♂, ECA, Amatole, Isidenge For. St., B1, 32.41 S, 27.14 E, 15 Nov 1987, E-Y 2515, *Podocarpus* bark, Endrody-Younga (TMSA); PT 1♂, MPU, Belfast, 6 Dec 1988, H.P. Terblanche (TMSA); PT 1♂, ECA, Kubusie Forest, Stutterhaim, C.P., 3 Jan 1980, N.J. Duke (TMSA); PT 1♂, ECA, Buffalo Pass E.L., 20 Dec 1979, N.J. Duke (TMSA); PT 1♂, ECA, Buffalo Pass E.L., 23 Oct 1979, N.J. Duke (TMSA); PT 1♂, ECA, East London, 29 Oct 1923, G. van Son (TMSA); PT 1♂, ECA, P. Elizabeth, 6 Jan 1910, on beach (TMSA); PT 1♂, ECA**,** G. town, Oct 1894, 7.Pym (TMSA); PT 1♂, GAU, Pretoria, Waterkloof, 25.43 S, 28.11 E, 5 Nov 1989, Endrody-Younga (TMSA); PT 1♀, ECA, Van Staden’s Riv, near Thornhill, 33.55 S, 25.12 E, 06 Dec 1988, B. Grobbelaar (SANC); PT 1♀, ECA, Clark’s Siding 1650 m, Dordrecht 31 24 S, 27 07 E, 21 Dec 2000, B.H. Catherine (SANC); PT 1♀, KZN, Royal Natal National Park, Natal Drakensberg, 1550 m asl, Tugela Gorge, Montane *Podocarpus* forest, in old log on the ground, 21 Oct 1993, Pajor Istvan (SANC); PT 1♀, ECA, Pt. Elizabeth, 1899, J.L. Drege (SANC); PT 1♀, MPU, Mariepskop, Blyderivier, D. Wessels (SANC); PT 1♀, LIM, Blouberg 1480 m, 23 05 20 S, 29 01 60 E, 04–07 Dec 1990, Chown, Steenkamp & McGeogh (SANC); PT 1♂, ECA, East London CP, Sep 1923, Ent. SN. 2649 (SANC); PT 1♂, KZN, Oribi Gorge, Oct 1993, M. Vogt (SANC); PT 1♂, GAU, Heidelberg, 26.31 S, 28.12 E, 25 Nov 1984, R. Oberprieler (SANC); PT 3♂, GAU, Kempton Park, 26.06 S, 28.15 E, from larva in rotten wood, Nov 1987, P. Wight (SANC); PT 1♂, GAU, Johannesburg TP., Dec 1953, G.A. Hepburn (SANC); PT 3♂, GAU, Centurion Lyttelton, 25 48 S, 28 11 E, 1460 m, 07 Nov 1999, A. Glanvill (SANC); PT 1♂, KZN, Cathkin Peak, Drakensberg Mnts, 28 46 S, 29 10 E, 1400 m, 18–20 Dec 1998, P.E. Reavell (SANC); PT 1♂, LIM, Lajuma Farm, Soutpansberg, 23 02 S, 29 20 E, 22 Nov 1997, R. Stals (SANC); PT 1♂, KZN, Pietermaritzburg, town bush, Malaise trap, Oct 1976, R. Miller (SANC); PT 1♂, ECA, Bedford, 30 Dec. 1994, R. Perissinotto & L. Clennell (RPC);PT 1♀, KZN, Natal Midlands, Howick, 29°29’S, 30°14’E, 12 Dec. 1989, O Bourquin (ERC); PT 1♂, ECA, Amatole, Isigende For., Stat. A1, 32° 41' S, 27° 16' E, 28 Nov. 1987, Endrody-Younga (ERC); PT 1♂, GAU, Pretoria, Waterkloof, 25°43'S, 28°11'E, 18 Dec. 1988, Endrody-Younga (ERC); PT 1♂, GAU, Florida, 15 Oct. 1976 (ERC); PT 1♀, KZN, Karkloof For., 29°18'S, 30°13'E, 1300 m, 4 December 1989, Endrödy-Younga & Klimaszew (ERC); PTs 3♀, ECA, Fort Fordyce, 18 Jan. 1998, R. Perissinotto L. Clennell (ERC); PT 2♂, KZN, Vernon Crookes, 30°16'S, 30°36'E, 28 Nov. 1998, R. Perissinotto & L. Clennell (ERC); PT 1♂, ECA, Cape Recife, 6 Dec. 1997, R. Perissinotto & L. Clennell (ERC); PT 1♂, ECA, Cape Recife, 17 Feb. 1996, R. Perissinotto & L. Clennell (RPC); PT 1♀, ECA, Alexandria Forest, 25 Sep. 1994, R. Perissinotto & L. Clennell (RPC); PT 1♂, ECA, Cathcart/Queenstown Road, 1 Dec. 1997, M. Burger (RPC); PT 1♂, ECA, Morgan Bay, 16 Oct. 1994, R. Perissinotto & L. Clennell (RPC); PT 1♂, ECA, Van Stadens Mouth, 20 Feb. 1994, R. Perissinotto & L. Clennell (RPC); PTs 1♂1♀, ECA, Near Bedford, 24 Nov. 1995, R. Perissinotto & L. Clennell (RPC); PT 1♀, ECA, Fort Brown, 29 Nov. 1996; R. Perissinotto & L. Clennell (RPC); PT 1♀, ECA, Woody Cape, 7 Oct. 1994, R. Perissinotto & L. Clennell (RPC); PT 1♂, ECA, 1 PT (♂), Fort Fordyce, 15 Feb. 1998, R. Perissinotto & L. Clennell (RPC); PTs 1♂1♀, KZN, Karkloof, 22–23 Jan 2000, R. Perissinotto & L. Clennell (RPC); PT 1♂, same data but 3 Feb 2002 (RPC); PTs 1♂1♀, MPU, Barberton, Makonjwa Mt. 1250 m, 27 Dec. 1995, P Stobbia (RPC); PTs 2♂1♀, KZN, Umthamvuna, 24 Oct. 2004, R. Perissinotto & L. Clennell (RPC); PTs 1♂1♀, KZN, Oribi Gorge, 13 Nov. 2004, R. Perissinotto & L. Clennell (RPC); PTs 3♀, KZN, near Impendle, 11 Dec. 2004, R. Perissinotto & L. Clennell (RPC); PTs 1♂1♀, KZN, Pongola Bush N.R., 19 Dec. 2005, R. Perissinotto & L. Clennell (RPC); PTs 2 ♂2♀, same data but Oct. 2006 (RPC); 1♂, KZN, Cavern Resort, 29 Oct 2006, MDTP 92288 Coll III (RPC); 1♀, ECA, near Adelaide, 27 Dec 2007, R. Perissinotto & L. Clennell (RPC); 2♂3♀, KZN, Monk’s Cowl, 31 Oct 2009, R. Perissinotto & L. Clennell (RPC).

SWAZILAND: PT 1♀, Mbabane, Sidwashini, 18 Nov 1990, N.J. Duke (TMSA).

### Stripsipher jansoni

*Stripsipher jansoni* (Fig. 1B) is a mountain grassland inhabitant and is currently known from a small number of high altitude localities (Figs 1D, F): from the Compassberg (2300 m) in the Eastern Cape to the Kwamandlangampisi Mountain (2266 m) near Dirkiesdorp, in Mpumalanga (Fig. 2). An exception to this, possibly due to an error in identification, appears to be the record from the Mbotyi Forest (G. van Son, specimen data label) which is a coastal locality of elevation not exceeding approximately 500 m

Larvae of this mountain dweller have been found under stones and in crevices among rocks, where they feed on accumulations of decomposing grass litter or herbivore dung (Ricchiardi et al. 2008). Particularly well utilized appear to be the pellets of Rock hyrax, *Procavia capensis*, locally known as “dassie”, which at these high altitudes attract not only *Stripsipher jansoni*, but also scores of larvae of other Cetoniinae species (e.g. *Xeloma* spp., *Rhinocoeta* spp., *Trichostetha* spp. *Rhabdotis* spp., *Leucocelis* spp. *Parelaphinis moesta*, *Hypselogenia geotrupina*) (Holm and Marais 1992, Holm and Perissinotto 2004, R.P. pers. observ.). Larvae from the Drakensberg (Cobham Nature Reserve) and the Kwamandlangampisi Mountain have been reared successfully in captivity, in environmental control rooms in Durban by R.P.

Reflecting its high altitude habitat, *Stripsipher jansoni* exhibits a delay in its adult activity, compared to *Stripsipher orientalis*. Records range from October to March, with peaks in December (48% of total) and January (24%). *Stripsipher jansoni* belongs to a species group within the genus that does not exhibit any feeding at the adult stage. Adults have been found crawling on the ground, flying low above the ground (10–50 cm) or resting on a variety of grasses. On one occasion, a female specimen was collected on yellow flowers of an unidentified shrub species on the Compassberg (R.P. pers. observ.), but it could not be established whether this was for the purpose of feeding or simply resting.

**Material examined.** SOUTH AFRICA: LT ♂ (*Stripsipher jansoni*), Natal, without additional data, (RNHL); HT♂ (*Stripsipher drakensbergi*), FST, Golden Gate N.P., 28 30 S, 28 31 E, I-1980, IC Sharp (SANC); PT 1♂ (*Stripsipher drakensbergi*), KZN, Drakensberg, 4-I-1925, D Kroom (ERC); PT 1♀ (*Stripsipher drakensbergi*), ECA, Embotyi Forest, Pondoland, 25/28-II-1957, G van Son (TMSA); PTs 3♂3♀ (*Stripsipher drakensbergi*), ECA, Compassberg, 2300 m, 15 Dec1997, R Perissinotto & L Clennell (ERC, RPC); 2♂, same data but 27 Dec 1997 (RPC); 2♂1♀, same data but 28 Dec 1997 (RPC); 1♀, KZN, Cobham Nature Reserve, 10 Oct 1998, R Perissinotto & L Clennell (RPC); 1♂, same data but 14 Dec 1998 (RPC); 7♂3♀, same data but 13 Dec 1999 (RPC); 1♂, same data but 5 Feb 2000 (RPC); 1♀, same data but 8 Dec 2002 (RPC); 3♂, KZN, Bulwer Mt, 5 Mar 2000, R Perissinotto & L Clennell (RPC); 3♂2♀, FST, Zastron, 6 Jan 2000, R Perissinotto & L Clennell (RPC); 1♀, FST, Golden Gate, 11 Jan 2002, R Perissinotto & L Clennell (RPC); 1♂ [partly decomposed carcass], FST, Harrismith, 14 Dec 2002, R Perissinotto & L Clennell (RPC); 2♀ [partly decomposed carcass], MPU, Kwamandlangampisi Mt., Dirkiesdorp, 17 Dec 2005, R Perissinotto & L Clennell (RPC); 2♂1♀, same data but Nov 2006 (RPC); 1♂, ECA, Lady Grey, 14 Jan 2005, R Perissinotto & L Clennell (RPC); 1♀, ECA, larva Witteberge Jan 2005, emerged Durban Oct 2005, R Perissinotto & L Clennell (RPC); 1♂, ECA, Kramberg, 27 Dec 2007, R Perissinotto & L Clennell (RPC); 2♂, ECA, Matatiele, 7 Dec 2008, R Perissinotto & L Clennell (RPC).

LESOTHO: 1♂, High Orange Valley, 1500 m, 1906, E. Haug leg. (MNHN).

## Larval morphology

### Third instar larvae of the genus *Stripsipher* (Figs 3–6)

Material: 5 last instar larvae of *Stripsipher orientalis* (South Africa, KZN Karkoof, 29°18'55"S, 30°15'04"E, 1300 m asl, 14 Feb 2004, in rotten suspended tree branch, R. Perissinotto & L. Clennell lgt); 5 last instar larvae of *Stripsipher orientalis* (South Africa, KZN Entumeni, 28°52'43"S, 31°18'36"E, 854 m asl, Feb 2004, in decomposing but standing tree trunk, R. Perissinotto & L. Clennell lgt);3 last instar larvae of *Stripsipher jansoni* (South Africa, KZN Cobham, 29°39'36"S, 29°23'02"E, 2560 m asl, 13 Dec 1999, underground, on plant detritus around rock boulder, R. Perissinotto & L. Clennell lgt).

**Figure 3.**
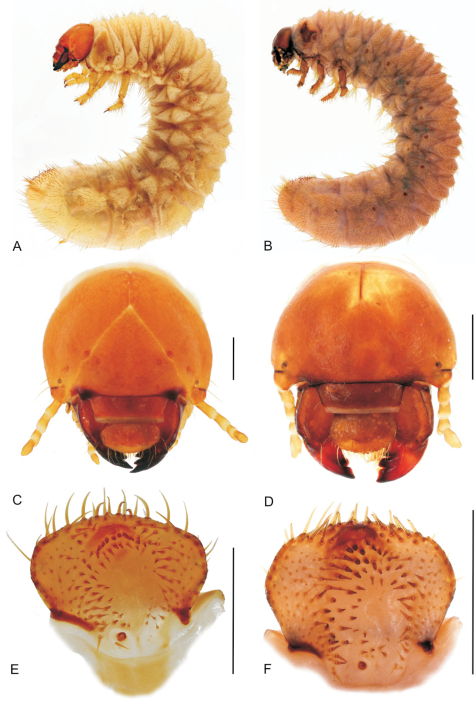
**A, B** habitus of fully grown larva (**A** – *Stripsipher orientalis* 51 mm **B** – *Stripsipher jansoni* 36 mm) **C, D** cranium of fully grown larva (**C** – *Stripsipher orientalis* **D** –*Stripsipher jansoni*) **E, F** Epipharynx (**E** – *Stripsipher orientalis* **F** – *Stripsipher jansoni*). Scale bar: 1 mm.

Larvae C-shaped, grub like, abdomen 10–segmented, segments of thorax and abdomen almost equal, abdominal segments 7–9 somewhat elongated. Length of larvae studied (third instars) 33–51 mm. **Head capsule** (Figs 3C, D):maximum width between 2.8 and 4.2 mm. Surface of cranium with faint microsculpture, orange brown to brown, antennifer, postclypeus and labrum brown. Cranial chaetotaxy summarized in Table 1. Frontal sutures straight, slightly crooked in the anterior part. Epicranial insertions of antennal muscles distinct, more sclerotised than the surrounding area of epicranium or only indicated as a small depression near middle of frontal sutures. Clypeus trapezoidal, anteclypeus narrow and membranous, postclypeus heavily sclerotised with one exterior and one anterior clypeal setae on each side. Frontoclypeal suture distinct. A single pigmented stemma present on each side.

**Table 1.** Cranial chaetotaxy of *Stripsipher orientalis* and *Stripsipher jansoni*.

	**epicranium**				**frons**				**clypeus**		**labrum**			
Group of setae	DES	PES	AES	EES	PFS	EFS	AFS	AAS	ACS	ECS	PLS	PMS	ELS	LLS
***Stripsipher orientalis***														
Long and medium setae	2(1)	1(2)	1	5–10	1	0	0	1	1	1	4–6	1	1	14–22
minute setae	0–2	2–5(0)	0 (1)	0–3	1	1	1	0	0	0 (1)	0-2	0 (1)	0	0
***Stripsipher jansoni***														
Long and medium setae	2	1–2	1	8–9	1	0	0	1	1	1	3–6	1	1	19–16
minute setae	0–2	0–3		0–1	0–1	0	0	0	0	0	0–2	0	0	0

Abbreviations: AAS = setae on anterior frontal angle; ACS = anterior clypeal setae; AES = anterior epicranial setae; AFS = anterior frontal setae; DES = dorsoepicranial setae; ECS = exterior clypeal setae; EES= exterior epicranial setae; EFS = exterior frontal setae; ELS = exterior labral setae; LLS = setae on lateral labral lobe; PES = posterior epicranial setae; PFS = posterior frontal setae; PLS = posterior labral setae; PMS = paramedial labral setae. Numbers in brackets indicate a rarely occurring state. For explanation of length categories of setae see ‘Material and methods’.

**Antennae** (Figs 3C, D): antennomeres (an) subequal in length (relative length: an I = an IV > an II > an III; an II about 2/3 of an I and an IV). Third antennomere (an III) with ventral, apical projection exhibiting a single sensory spot. Last antennomere (an IV) with two or three ventral and one dorsal sensory spot and a small apical sensory field with faint sensillae.

Labrum symmetrical, anterior margin convex with numerous setae. Medial part with transverse, emarginated protuberance. Dorsal labral surface with two transverse rows of setae. Posterior row with about 10 setae, anterior row with one paramedian and one lateral seta on each side.

**Figure 4.**
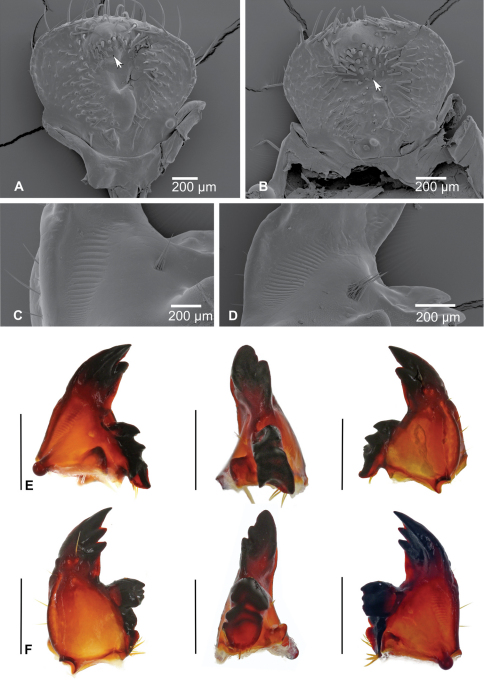
**A, B** SEM images of epipharynx, (**A** – *Stripsipher orientalis* **B** – *Stripsipher jansoni*) **C, D** right mandible, detail of the stridulatory area (**C** – *Stripsipher orientalis* **D** – *Stripsipher jansoni*) **E** – *Stripsipher orientalis*, right mandible: ventral, medial and dorsal aspect **F** – *Stripsipher orientalis*, left mandible: dorsal, medial and ventral aspect. Scale bar: 1mm (when not otherwise specified).

**Epipharynx** (Figs 3E–F, 4A–B): Haptomerum with strong haptomeral process and two to four transversal arched rows of stout setae. Haptomeral process prominent, well sclerotised with apical field of five pores and several others scattered around. Acroparia with about 30 long setae, clithra and epizygum absent. Lateral margin of epipharynx heavily sclerotised, especially in distal part. Plegmata and proplegmata absent. Acanthoparia with seven to eight setae. Chaetoparia asymmetric, with right exhibiting seven to eight irregular rows of setae and left five to six. Setae of medial rows stout, spine-like. Dexiotorma bar-like, straight, longer than laeotorma, right pternotorma almost or entirely absent. Laeotorma triangular with posterior pternotorma. Sense cone of haptolachus with four pores, sclerotised plate absent. Crepis faintly sclerotised, indistinct.

**Mandibles** (Figs 4C–F): prominent asymmetrical, scrobis with several setae and a deep longitudinal furrow. Two prominent setae and two pores present on apical half of dorsal mandibular surface, and one posterior seta at distal end of longitudinal furrow. Patches of dorsomolar and ventromolar setae present on both mandibles, ventromolar setae concealed in a single rim, dorsomolar setae more or less separated in the respective rims. Stridulatory area present with about 20 transversal ridges (Figs 4C, D). Right mandible with two and left mandible with three scissorial teeth. Molar lobes of both mandibles with sharp projections; posterior margin of calyx in medial aspect concave on right mandible, flattened and convex on left; brustiae with three to four and 10–12 setae on right and left mandible, respectively.

**Figure 5.**
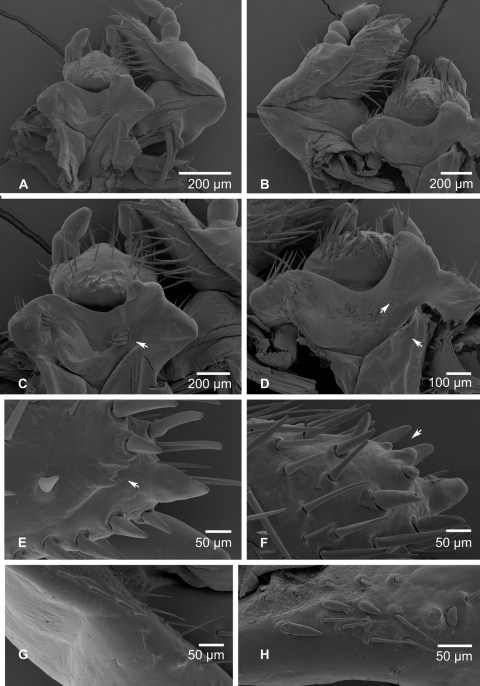
**A, B** Labio-maxillar complex and hypopharynx, dorsal aspect **(A** – *Stripsipher orientalis* **B** – *Stripsipher jansoni*) **C, D** Labium and hypopharynx, SEM image (**C** – *Stripsipher orientalis* **D** – *Stripsipher jansoni*) **E, F** apex of maxilla, unci, latero medial aspect (**E** – *Stripsipher orientalis* **F** – *Stripsipher jansoni*) **G, F** stridularory teeth of maxilla (**G** – *Stripsipher orientalis* **F** – *Stripsipher jansoni*).

**Figure 6.**
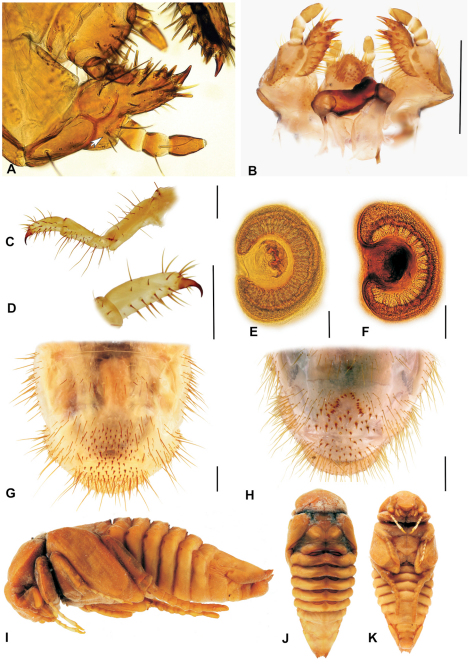
**A** *Stripsipher orientalis*, labium and maxilla, ventral aspect (arrow indicating basal joint of maxillar palpus retracted into palpiger) **B** – *Stripsipher jansoni*, labiomaxillar complex, dorsal aspect **C, D** *Stripsipher orientalis*, metathoracic leg **C** – general view, lateral aspect, **D**–metatibiotarsus with claw; **E, F** – thoracic spiracles (**E** – *Stripsipher orientalis* **F** – *Stripsipher jansoni*) **G, H** last abdominal segments, ventral aspect (**G** – *Stripsipher orientalis* **F** – *Stripsipher jansoni*) **I, J, K** *Stripsipher jansoni* pupa, lateral, dorsal, and ventral aspect (14.8 mm). Scale bar: 1 mm.

**Maxilla** (Figs 5, 6A, B): dorsal surface of cardo and dorsal and ventral surfaces of labacobaria with hair-like setae and pores; vertral side of cardo bald. Dorsal surface of stipes with 10–20 slender hair-like setae in one to three longitudinal rows; oblique row of two to six well sclerotised spine-like stridulatory teeth and an anterior truncate process (blunt tubercle) distad to the row (Figs 5A, B, G, H, 6B). Stipes with few ventral setae. Galea and lacina entirely fused forming mala, galeo-lacinial suture dorsally visible as desclerotised line (Fig. 6B), entirely absent on ventral face (Fig. 6A). Galean portion of mala with single falcate uncus and several long and stout hair-like setae in longitudinal rows; lacinial part of mala with two reduced unci fused at their base and two apical stout spine-like setae (Figs 5E–F); dorsomedial side with numerous and very long hair-like setae. Ventral surface of mala with row of three to five stout setae and few long setae. Maxillary palps four-jointed, with basal joint often partially retracted into the palpifer, making palps appear three-jointed (Figs 5A, B, 6A, B). Penultimate joint usually with two setae.

**Hypopharyngeal sclerome** (Figs 5C, D) asymmetrical with strong protruding and pointed truncate process. Tufts of tegumentary expansions (= phoba, sensu Böving, 1936) present on left lateral lobe, hypopharyngeal sclerome proximal to truncate process and below hypofaryngeal sclerome, but present or absent near its right proximal border. Right lateral lobe not sclerotised.

**Ligula** (Figs 5C, D) dorsallywith numerous setae, proximal setae stout conical and short, posterior setae hair-like, long. Dorsal surface of ligula with pit-like organ in the medial portion and several pores in the distal half. Posterior margin with pair of long paramedian setae. Labial palpi two-segmented.

**Thorax** (Figs 3A, B): Prothorax with single dorsal lobe, meso- and methatorax with three well developed lobes. Each dorsal sublobe of thoracic segments with one to three rows of short setae, posterior row with short setae interspersed with long setae. Prothoracic sclerite covering almost the whole lateral portion of prothorax, posterior border with median projection extending almost behind the distal margin of mesothoracic spiracle. Mesothoracic spiracle (Figs 6E, F) with C-shaped respiratory plate; distance between lobes of respiratory plate almost equal to the maximum diameter of respiratory plate. Respiratory plate with 12–25 holes across diameter. All pairs of legs (Figs 3A, B, 6C, D) subequal and with similar tarsal claws; claws falcate, sharp pointed and bearing two proximal setae (Fig. 6D).

**Abdomen** (Figs 3A, B, 6G, H):ten-segmented. Dorsa of abdominal segments I–VI with three sublobes, segments VII and VIII with only two. Each sublobe bearing three to five rows of short setae, posterior one or two rows with long setae interspersed between the short ones. Abdominal spiracles similar to mesothoracic spiracles, but slightly smaller. Size of spiracles decreasing caudad. Smallest spiracle on abdominal segment VIII, reaching about two thirds of the size of the mesothoracic one. Raster either without palidium and with fused teges composed of numerous hamate setae (septula absent, Fig. 6G), or with palidium consisting of paired oblique rows of pali (Fig. 6H). Each row of approximately ten pali, extending forward and inwards, thus forming a “V” or “U” on the medial portion of last abdominal segment, enclosing a small septula. Teges composed of few hamate setae. Ventral anal lip with 20–40 hamate setae, dorsal lip with several long hair-like setae; same setae present also on ventrolateral and ventroanterior portions of last abdominal segment.

### Morphology of pupa (Figs 6I–K)

Length 14.8–19.4 mm, maximal width 6–8.2 mm. Exarate, testaceous, surface glabrous. Head bent ventrally. Mouthparts and antenna well separated. Labrum tumid, clypeus slightly concave. Maxilla elongated and conical. Compound eyes distinct. Thorax: pronotal disk convex. Lateral margins of pronotal disc distinct. Meso- and metanotum differentiated. Mesonotum with triangular posterior projection. Pterotecae free, closely compressed around body and almost equal in length. Spines and spurs on tibiae rudimentary, tarsomeres well defined. Abdomen: dorsally with nine visible, progressively narrowing segments. Terga of segments I–VI with five pairs of gin traps (*sensu* Hinton 1946) or dioneiform organs (*sensu* Costa et al. 1988, Fig. 6J). First pair heavily sclerotised, with straight or slightly concave anterior and concave posterior margin. Subsequent gin traps less sclerotized, sickle-like, convex on both margins. Tergum of first abdominal segment with transversal row of protuberances ahead of gin traps. Spiracles on first four abdominal segments functional, with sclerotized ring, first spiracular pair covered by pterothecae. Spiracles on abdominal segments V–VIII non-functional and rudimentary. Last abdominal segment with pair of small, pointed, non-articulated urogomphi. Genital ampula of male pupae spherical and prominent.

**Diagnostic characters of *Stripsipher orientalis* Ricchiardi, 2008** (Figs 3A, C, E; 4A, C, E, F; 5A, C, E, G; 6A, C–E, G).

Material: five last instar larvae (South Africa, KZN Karkoof, 29°18'55"S, 30°15'04"E, 1300 m asl, 14 Feb 2004, in rotten suspended tree branch, R. Perissinotto & L. Clennell lgt.); five last instar larvae (South Africa, KZN Entumeni, 28°52'43"S, 31°18'36"E, 854 m asl, Feb 2004, in decomposing but standing tree trunk, R. Perissinotto & L. Clennell lgt).

Body length 34–51 mm, cranium width between 3.9–4.2 mm (n = 5). Chaetotaxy as in table 1. Antenna with 3 ventral sensory spots (n = 9; in a single case only two ventral sensory spots present). Haptomerum of epipharynx with two transversal arched rows of 12–13 (7–9) stout setae respectively and a proximal sclerotised area (Figs 3E, 4A). Acanthoparia asymmetric, right half with approximately 55–60 setae, left with 40–45. Maxilla: stipes dorsal with a single row of approximately 10 slender hair-like setae and two to four narrow stridulatory teeth (Fig. 5G). Ventral face of mala with a row of four to six stout conical setae. Lacinial unci pointed (Fig. 5E). Tufts of tegumentary expansions (= phoba, *sensu* Böving, 1936) on the hypopharyngeal sclerome proximal to truncate process present, but absent below the hypopharyngeal sclerome (near to its right proximal border) (Fig. 5C). Dorsal part of ligula with approximately 40 conical to long hair-like setae (Fig. 5C). Size of thoracic spiracles 0.42–0.43 x 0.3–0.32 mm (length x width). Respiratory plate with 20–25 holes across diameter (Fig. 6E). Body vestiture more or less sparse; setae on dorsum of ninth abdominal segment separated by bald area into two fields. Raster without palidium and septula, teges fused (Fig. 6G). Hamate setae of teges subequal in size and shape.

**Diagnostic characters of *Stripsipher jansoni* Péringuey, 1908** (Figs 3B, D, F; 4B, D, F; 5B, D, F, H; 6B, F, H).

Material: three last instar larvae; two pupae (South Africa, KZN Cobham, 29°39'36"S, 29°23'02"E, 2560 m asl, 13 Dec 1999, underground, on plant detritus around rock boulder, R. Perissinotto & L. Clennell lgt).

Body length 33–36 mm, cranium width 2.7–2.8 mm (n = 3). Chaetotaxy as in table 1. Antenna with 2 ventral sensory spots. Haptomerum of epipharynx (Figs 3F, 4B) with three to four transversal rows of four to eight stout setae, proximal sclerotised area absent. Acanthoparia asymmetric; right half with approximately 55–62 setae, left with 40–50. Maxilla (Figs 5B, 6B): stipes dorsal with one or two rows of approximately 10–18 slender hair-like setae and two to six conical stridulatory teeth (Fig. 5H). Ventral face of mala with row of three stout conical setae (occasionally another stout seta preceding the row, reaching about ½ of the size of the following three), lacinial unci obtuse. Ligula dorsal with 50–60 short conical to long hair-like setae (Fig. 5D). Size of thoracic spiracle 0.35–0.40 × 0.24–0.26 mm (length x width). Respiratory plate with 12–16 holes across diameter. Body vestiture more or less dense, setae on dorsum of ninth abdominal segment not separated by bald area into two fields. Palidium of raster consisting of paired oblique rows (Fig. 6H). Each row of approximately ten pali, extending forward and inwards thus forming an inverted “V” or “U” shaped row on the medial portion of last abdominal segment, enclosing a small septula. Teges composed of few hamate setae. Two distinct types of hamate setae present on teges, small and large with broadened base. Large hamate setae about three times longer than pali.

## Discussion

### Comparison of *Stripsipher* with other Trichiinae larvae

Currently, information of immature stages of Trichiinae (sensu Krikken 1984) is only available for 38 species in 15 genera (including the tribes Cryptodontini, Incaini, Osmodermatini, Trichiini; Šípek in press). Beside the herein described two species, only larvae of *Clastocnemis quadrimaculatus* (Afzel, 1817), *Platygenia barbata* (Afzel, 1817), *Coelocorynus desfontainei* Antoine, 1999 and *Stripsipher opacicauda* Arrow, 1926 are know from sub-Saharan Africa (Jerath and Unny 1965; Šípek et al. 2009). Since it has been suggested that the Trichiinae may be paraphyletic (Micó et al. 2008, Šípek et al. 2009), it is important at this stage to discuss the differences of immature *Stripsipher* with those known from other Trichiinae genera.

Larvae of *Osmoderma* and *Platygenia* have fused abdominal segments IX and X, which is in contrast to what is found in all other known Trichiinae, which have a ten-segmented abdomen. Moreover, larvae of *Osmoderma* lack visible stemmata and the shape of their tarsal claw also differs significantly from that of all other Trichiinae.

**Figure 7.**
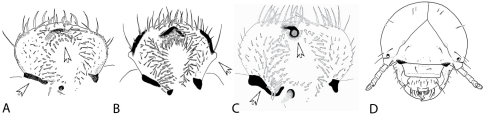
**A–C** morphology of epipharynx: **A** – *Stripsipher orientalis* **B** – *Coelocorynus desfontainei* (from Šípek et al. 2009) **C** – *Gnorimus nobilis* **D** – *Stripsipher orientalis*, schematic diagram of cranial chetotaxy.

The morphology of the epipharynx is amongst the most critical characters separating *Stripsipher* from all other non-African Trichiini (e.g. Medvedev 1952, Ritcher 1966, Morón 1983, Zhang 1984, Delgado-Castillo and Morón 1991, Sawada 1991, Morón 1995, Klausnitzer and Krell 1996). In *Stripsipher* (as well as in *Clastocnemis* and *Coelocorynus*)the haptomeral process is located distad to a transverse row of stout setae and the proximal end of dexiotorma is straight (Figs 7A, B). In all the remaining Trichiinae, on the other hand, the row of haptomeral setae is interrupted by a strong process and the dexiotorma is bent mesally at its proximal end (Fig. 7C).

### African Trichiinae

As stated above, larvae of only four genera of Trichiinae are known from sub-Saharan Africa. Apart from body size, *Platygenia* larvae can also be separated from the rest by the fusion of abdominal segments IX and X. Larvae of *Coelocorynus* are characterized by strong lateral tubercles on epipharynx (Fig. 7B) and a drastic reduction of lacinial unci. The larvae of *Clastocmenis quadrimaculatus* seem to be the most similar to the larvae of *Stripsipher*. However, the description provided by Jerath and Unny (1965) is incomplete, so the only difference that can be derived from this lies in the number of lacinial unci. While in *Clastocnemis* there is a single lacinial uncus, two small unci can be observed in *Stripsipher* larvae.

### Phylogenetic relationships

**Figure 8.**
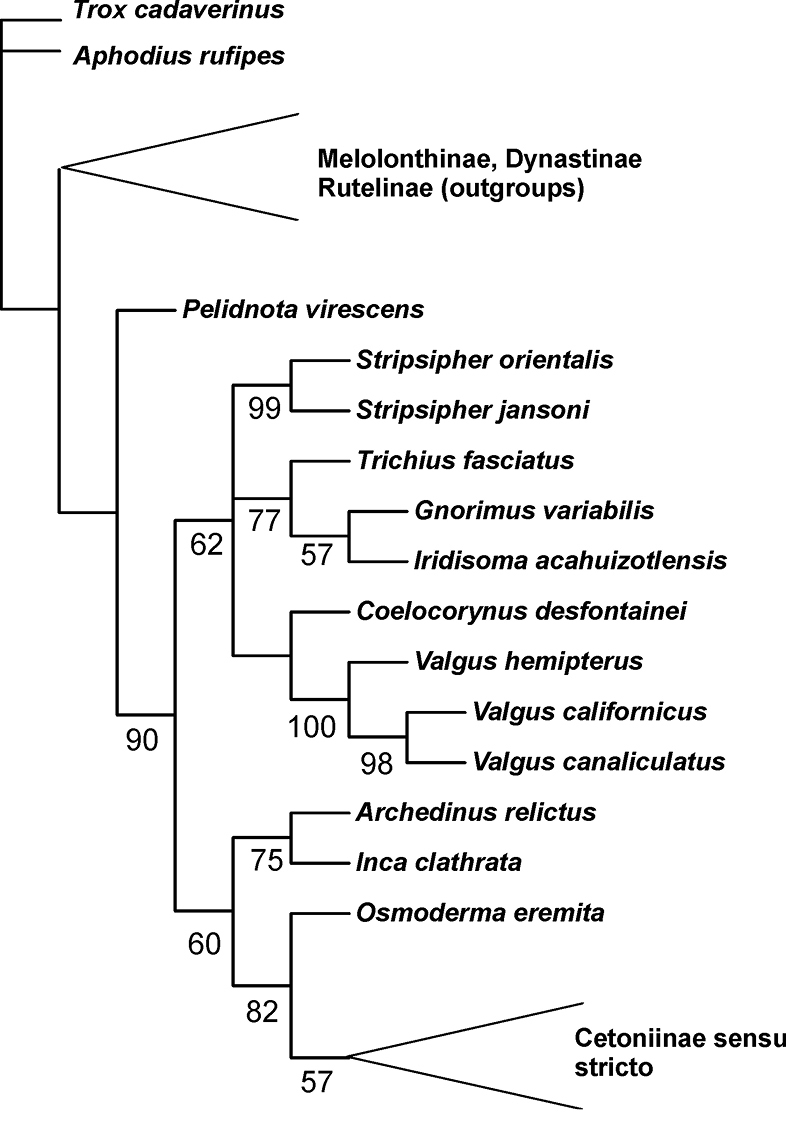
Phylogenetic position of the genus *Stripsipher*, based on the morphological data matrix of Micó et al. (2008) and Šípek et al. (2009).

To evaluate the phylogenetic relationships of the genus *Stripsipher*, both larval and adult morphological characters of the two species were coded into a data matrix used in previous phylogenetic studies of the Cetoniinae (Micó et al. 2008, Šípek et al. 2009). Not surprisingly, results do not differ significantly from those obtained in previous works. However, the genus *Stripsipher* proved to be firmly nested within the Trichiini-Cryptodontini-Valgini clade (Fig. 8). This finding supports the empiric observation that the larvae of *Stripsipher* strongly resemble the known larvae of all other Trichiinae, with the exception of those belonging to the genera *Osmoderma*, *Inca*, *Archedinus* and, so far as can be derived from the brief description in Jerath and Unny (1965), also of the genus *Platygenia*. Regardless of the controversial position of the genus *Valgus* within a clade comprised of Trichiini and Cryptodontini members, this study supports once again the hypothesis that the Trichiinae (*sensu* Krikken, 1984) represent a paraphyletic group (see also Browne and Scholtz 1998; Micó et al. 2008; Smith et al. 2006).

## References

[B1] Bouchard P Bousquet Y Davies AE Alonso-Zarazaga MA Lawrence JF Lyal CHC Newton AF Reid CAM Schmitt M Ślipiński SA Smith ABT (2011) Family-group names in Coleoptera (Insecta). ZooKeys 88: 1-972. doi: 10.3897/zookeys.88.807 PMC3088472 21594053

[B2] Böving AG (1936) Description of the larva of *Plectris aliena* Chapin and explanation of new terms applied to the epipharynx and raster. Proceedings of the Entomological Society of Washington 38, 169–185.

[B3] Browne J Scholtz CH (1998) Evolution of the scarab hind wing articulation and wing base: a contribution towards the phylogeny of the Scarabaeidae (Coleoptera: Scarabaeoidea). Systematic Entomology 23: 307-326. doi: 10.1046/j.1365-3113.1998.00059.x

[B4] Delgado-Castillo L Morón MA (1991) A new genus and species of Trichiini from Mexico (Coleoptera: Melolonthidae). Pan-Pacific Entomologist 67: 181-188.

[B5] Hayes WP (1929)Morphology, taxonomy and biology of larval Scarabaeoidea. Illinois Biological Monographs 12: 88-200.

[B6] Holm E Marais E (1992) Fruit Chafers of Southern Africa (Scarabaeidae: Cetoniini). Ekogilde CC, Hartebeespoort, 326 pp.

[B7] Holm E Perissinotto R (2004) New and lesser known species of African fruit chafers (Coleoptera Scarabaeidae Cetoniinae). Tropical Zoology 17: 73-95.

[B8] Jerath ML Unny KL (1965) Larvae of six genera of Cetoniinae from eastern Nigeria (Coleoptera: Scarabaeidae). The Coleopterists Bulletin 19: 59-64.

[B9] Klausnitzer B Krell FT (1996) 6. Überfamilie Scarabaeoidea. In: Klausnitzer B (Ed.). Die Käfer Mitteleuropas, Bd. L3: Die Larven der Käfer Mittteleuropas. 3. Band. Polyphaga. Teil 2. Goecke & Evers, Krefeld: 11-89.

[B10] Krikken J (1984) A new key to the suprageneric taxa in the beetle family Cetoniidae, with annotated lists of the known genera. Zoologishe Verhandelingen 210: 1-77.

[B11] Medvedev SI (1952) Lichinki plastinschatousych zhukov fauny SSSR. Opredeliteli po faune SSSR 47. [Larvae of the Scarabaeoidea (Coleoptera) of the Soviet Union. Keys to the Identification of the Fauna of USSR 47]. Izdatelbstvo Akadmii Nauk SSSR, Moscow-Leningrad, 342 pp.

[B12] Micó E Morón MA Šípek P Galante E (2008) Larval morphology enhances phylogenetic reconstruction in Cetoniidae (Coleoptera: Scarabaeoidea) and allows the interpretation of the evolution of larval feeding habits. Systematic Entomology 33: 128-144.

[B13] Morón MA (1983) Los estados inmaduros de *Inca clathrata sommeri* Westwood (Coleoptera, Melolonthidae, Trichiinae): con observaciones sobre el crecimiento alometrico del imago. Folia Entomologica Mexicana 56: 31-51.

[B14] Morón MA (1995) Larva and pupa of *Archedinus relictus* Morón and Krikken (Coleoptera: Melolonthidae, Trichinae, Incaini). Pan-Pacific Entomologist 71: 237-244.

[B15] Péringuey L (1908) Descriptive catalogue of the Coleoptera of South Africa. Additions and corrections. Transactions of the South African philosophical Society 13: 682-683.

[B16] Ricchiardi E (1998) Notes for the revision of the genus *Stripsipher* Gory & Percheron, 1833, with description of four new species (Coleoptera, Cetoniidae, Trichiinae, Trichiini). Mitteilungen der Münchner Entomogischen Gesellschaft 88: 45-64.

[B17] Ricchiardi E Perissinotto R Clennell L (2008) Taxonomic revision of the South African genus *Stripsipher*, with description of four new species (Coleoptera Cetoniidae). Bollettino della Societá Entomologica Italiana 140: 155-178.

[B18] Ritcher PO (1966) White Grubs and their Allies. A study of North American Scarabaeoid Larvae. Oregon State Monographs. Studies in Entomology Nr. 4. Oregon State University Press, Corvallis, 219 pp.

[B19] Sawada H (1991) Morphological and phylogenetical study on the larvae of Pleurostict Lamellicornia in Japan. Tokyo University of Agriculture Press, Tokyo. ii + 132p +157 plates + ii.

[B20] Scholtz CH Grebennikov VV. (2005) 12. Scarabaeiformia Crowson, 1960. 13. Scarabaeoidea Latreille, 1802. In: Beutel RB, Leschen RAB (Eds) Handbuch der Zoologie / Handbook of Zoology, (4)38, 345–425.

[B21] Smith ABT Hawks DC Heraty JM (2006) An overview of the classification and evolution of the major scarab beetles clades (Coleoptera: Scarabaeoidea) based on preliminary molecular analyses. Coleopterists Society Monograph 5: 35-46. doi: 10.1649/0010-065X(2006)60[35:AOOTCA]2.0.CO;2

[B22] Šípek P Grebennikov VV Gill B (2009) Afromontane *Coelocorynus* (Coleoptera: Scarabaeidae: Cetoniinae): larval descriptions, biological notes, phylogenetic analysis and polyphyly of Trichiini. European Journal of Entomology 106: 95-106

[B23] Šípek P Král D (in press) Immature stages of the rose chafers (Coleoptera: Scarabaeidae: Cetoniinae): an historical overview. Zootaxa.

[B24] Šípek P Král D Jahn O (2008) Description of the larvae of *Dicronocephalus wallichi bourgoini* (Coleoptera: Scarabaeidae: Cetoniinae) with observations on nesting behavior and life cycle of two *Dicronocephalus* species under laboratory conditions. Annales de la Société Entomologique de France 44: 409-417.

[B25] Švácha P Danilevsky ML (1986) Cerambycoid Larvae of Europe and Soviet Union (Coleoptera, Cerambycoidea). Part I. Acta Universitae Carolinae – Biology 30: 1-176.

[B26] Zhang ZL (1984) Coleoptera: Larvae of Scarabaeoidea. Economic Insect Fauna of China 28: 1-107.

